# An introduction to ghost imaging: quantum and classical

**DOI:** 10.1098/rsta.2016.0233

**Published:** 2017-06-26

**Authors:** Miles J. Padgett, Robert W. Boyd

**Affiliations:** 1 School of Physics and Astronomy, University of Glasgow, Glasgow G12 8QQ, UK; 2 Department of Physics, and School of Electrical Engineering and Computer Science, University of Ottawa, Ottawa, Ontario, K1N 6N5, Canada; 3 The Institute of Optics, and Department of Physics and Astronomy, University of Rochester, Rochester, NY 14627, USA

**Keywords:** ghost imaging, quantum imaging, parametric down-conversion

## Abstract

Ghost imaging has been a subject of interest to the quantum optics community for the past 20 years. Initially seen as manifestation of quantum spookiness, it is now recognized as being implementable in both single- and many-photon number regimes. Beyond its scientific curiosity, it is now feeding novel imaging modalities potentially offering performance attributes that traditional approaches cannot match.

This article is part of the themed issue ‘Quantum technology for the 21st century’.

## 1. Introduction

‘Ghost imaging’ is often understood as imaging using light that has never physically interacted with the object to be imaged. Instead, one light field interacts with the object and a separate light field falls onto the imaging detector. Ghost imaging functions by means of the spatial correlations between these two beams. At low photon numbers, these correlations are usually created as a direct consequence of the process of parametric down-conversion, in which a single incoming pump photon creates a pair of photons, termed signal and idler photons, which are strongly correlated in position. However, despite the quantum origin of its initial demonstration [1], it was realized that many of the functionalities of a ghost imaging system could be demonstrated using classical correlations [2,3]. In a typical classical ghost imaging scenario, where the photon number is much higher, a simple beam splitter creates a copy (duplicating both intensity and phase) of a spatially structured beam where the fidelity of the copy is limited only by the Poissonian statistics of the photon numbers in the two beams.

## 2. Ghost imaging using correlated photons

In the 1990s, Shih with co-workers and others published a series of papers showing how spatial correlations between signal and idler photon pairs produced by parametric down-conversion could be used within a ghost imaging system [1,4,5]. Within these systems, the image information is not present in either the signal or the idler photons alone, but rather is revealed only in the correlations between them [6]. The fundamental idea behind this approach to imaging, which has come to be known as ghost imaging, is that the output plane of the nonlinear crystal in which the down-conversion occurs is imaged such that the idler (or signal) photons are imaged onto the object and the signal (or idler) photons are imaged onto a detector array, or more often a raster-scanned detector.^1^ An additional, large-area, yet single-element (single-pixel) detector, often known as the bucket detector, collects the photons transmitted or backscattered by the object, and thus this single-pixel detector records no image information, but rather gives a binary output depending on whether the photon was transmitted/backscattered or not. Similarly, the imaging detector gives no direct image information about the object, but rather after many photons are recorded gives simply the cross-sectional profile of the down-conversion emission. The ghost imaging protocol entails recording the coincidence counts between the signal and idler photons. Specifically, the position of the photon hitting the imaging detector is recorded only if its detection is coincident with the recording of a photon by the other detector. In essence, the idler photons have illuminated the object, and the signal photons, which are correlated with the idler photons, have been recorded by the imaging detector. The subset of those position-measured signal photons that is coincident with detection of an idler photon reveals the image.

## 3. The Klyshko model for ghost imaging

**Figure 1.**
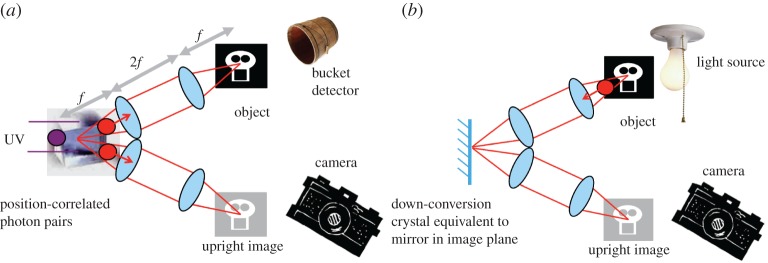
The images produced by a ghost imaging system (*a*) based on spontaneous parametric down-conversion (SPDC) are equivalent to those that could be produced by a classical imaging system (*b*), albeit the ghost imaging system has a different time sequence of events.

During the early work on ghost imaging, Klyshko established an intuitive model from which the outcome of such an experiment could be predicted [7]. In this Klyshko, or ‘back-projection’, model the single-element detector is conceptually replaced with a light source and the nonlinear crystal is replaced with a mirror. Within the equivalent to a standard ghost imaging configuration, we see now that the light source illuminates the object, the plane of which is imaged to the crystal/mirror, from which the light is reimaged to the imaging detector. Hence light emitted from the source illuminates the object and this object plane is imaged, firstly to the crystal/mirror and then reimaged to the imaging detector (figure 1).

For conventional imaging systems, the degree of spatial coherence in the illumination light can have some subtle effects on the details of the image, but both coherent and incoherent illumination give rise to largely similar images. However, when seeking to observe the diffraction pattern of an object, the spatial degree of coherence of the illumination light is paramount, and ghost imaging systems are similarly dependent upon spatial coherence [4]. Within the Klyshko model, we can see that in order to observe the diffraction pattern we would have to both illuminate the object with spatially coherent light and move the imaging detector into the far field of the crystal/mirror, i.e. the far field of the object [8]. This spatially coherent illumination in the Klyshko model corresponds to spatially coherent detection in the ghost imaging system, i.e. restricting the aperture of the single-element detector such that the photons it detects are in a single spatial mode. This mode selectivity is readily achieved by coupling the single-element detector through a single-mode optical fibre. Although significantly reducing the optical flux, this spatial selectivity applied to the idler photons means that the signal photon is similarly conditioned and the resulting ghost diffraction pattern is of high contrast.

## 4. Ghost imaging using correlations derived from quantum entanglement

The use of parametric down-conversion to produce the photon pairs commonly used in ghost imaging systems might tempt one to conclude that ghost imaging is a fundamentally quantum process. Indeed, it is well established that parametric down-conversion leads to the creation of entangled photon pairs. However, the process of ghost imaging makes use only of the spatial correlation of the photon pairs, and spatial correlation is a property that could, in principle, be derived from a classical source [3,9]. Indeed a scanning laser beam incident on a beam splitter allows the object to be probed with one while simultaneously obtaining knowledge of the position of the scan with the other beam. Such a wholly classical system can recreate many of the image attributes of a down-conversion source [2,10].

**Figure 2.**
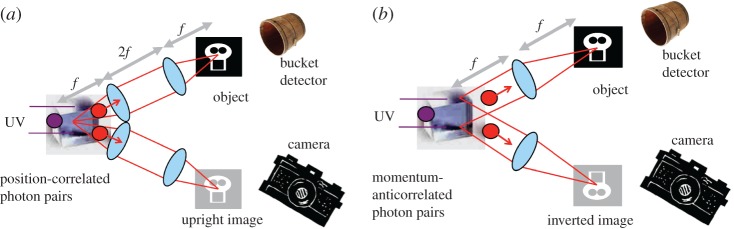
Ghost imaging systems can be configured to take advantage of either the position (*a*) or momentum (*b*) correlations inherent in SPDC. The fact that one can do either is a manifestation of EPR-type correlations.

We recall that in the context of Einstein–Podolsky–Rosen (EPR) studies, one needs to demonstrate correlations in each of two complementary variables, such as correlation in both position and transverse momentum of the two photons [11]. In the imaging system described above, the plane of the crystal is imaged to both the object and the imaging detector, relying upon the near-perfect spatial correlations between signal and idler photons in the down-conversion process in order to form an image. However, in the plane of the crystal, the signal and idler photons are anticorrelated in their transverse momentum. Consequently, in the far field of the crystal, the positions of the signal and idler photons are anticorrelated. Placing both the object and the imaging detector in the far field of the crystal therefore results in a ghost imaging system, but one in which the image is now inverted with respect to the object. This upright or inverted image, depending upon the system configuration, is a manifestation of EPR entanglement (figure 2), and although both results could be independently produced using scanning laser beams, no single classical system could produce both [12,13].

## 5. Benefits of ghost imaging

Another embodiment of ghost imaging that offers special performance is when parametric down-conversion is phase-matched to give non-degenerate signal and idler wavelengths [14]. In this way, the object can be illuminated at one wavelength and the spatial information recorded at another, perhaps where the imaging detector is more sensitive or less noisy. Nearly all ghost imaging systems demonstrated to date have suffered from the same practical flaw, namely that the imaging detector is a raster-scanned, single-element detector, and this fact massively reduces the overall effective quantum efficiency of the system in proportion to the number of pixels in the image. Ideally, one would wish to use a detector array as the imaging detector; however, until recently the cumulative noise of such arrays was too high to measure reliably the position of a single photon within the field of view. One contribution from our groups [12,14,15] has been to use an intensified camera to do exactly this. By reducing the gate time of the intensifier such that the gain is large only for the instant of the photon arrival, it is possible to reduce the number of dark events to far fewer than one dark event per frame. The detection of the idler photon by the single-element detector is used to gate the detection of the position-correlated signal photon, and summing over many photon events reveals the image. The time sequencing between idler and subsequent signal detection, further lengthened by the trigger delay of the camera itself, means that in our configuration the signal photons need to be delayed using an image-preserving optical delay line. This camera-based approach has recently been configured in a microscope, taking advantage also of the non-degenerate phase matching to give infrared illumination, yet visible detection of microscopic objects [14].

In recent work by Zeilinger and co-workers [16] an alternative imaging configuration has also shown similar wavelength transforming capabilities. Interference between the outputs of simultaneously pumped down-conversion crystals creates a situation where blocking or changing the phase of one of the idler beams changes the interference between the visible beams, thus creating an image. Note that the idler beam, the only beam to interact with the object, does not itself even need to be detected.

A further variant of ghost imaging is quantum-secured imaging, which ensures that the image is a true representation of the object. It rules out the possibility that an eavesdropper, or the object itself, has manipulated the photons directed towards the observer in such a way that the image has been falsified. A process similar to the BB84 protocol of quantum key distribution can be used to ensure the integrity of the imaging process [17]. In the language of quantum communication Alice (the sender) and Bob (the receiver) constitute the imaging system, and are hence located in the same location, whereas the object to be imaged plays the role of the eavesdropper. The eavesdropper could steal photons from the illumination beam and replace them with photons with a transverse structure of a falsified object. To guard against such tampering, Alice and Bob use polarization encoding on single-photon transmissions and standard quantum communication protocols to ensure that the photons received are actually those that were sent.

In other ghost imaging configurations, one can record the image of both the image and idler beams such that their ratio suppresses the Poissonian nose inherent in both, giving noise-suppressed imaging [18]. Indeed, the latest generation of camera technologies allows observation of entanglement over many thousands of spatial modes [19].

## 6. Image denoising with low photon numbers

The ability to record the spatial position of individual photons raises the question as to exactly how many photons does it take to form an image. Typically images taken with a high-performance digital camera might contain as many as 10^5^ photons per pixel. At the other extreme, it is possible to distinguish optical modes from one another using a single photon; see, for example, the work on the single-photon measurement of orbital angular momentum [20]. The key to distinguishability is the degree of orthogonality between the images/states one wishes to distinguish. The ability to distinguish images is reduced because the phase is often inaccessible for measurement; one can only measure intensity distributions. However, the intensity distributions of two orthogonal images are not, in general, orthogonal, and consequently multiple photons are needed to distinguish one image from the other. When the average number of photons per pixel is low, then even in images where adjacent pixels have similar underlying intensities the numbers of photons in adjacent pixels are likely to be different due to the Poissonian nature of shot noise. Consequently, images comprising a small number of photons are inherently noisy.

**Figure 3.**
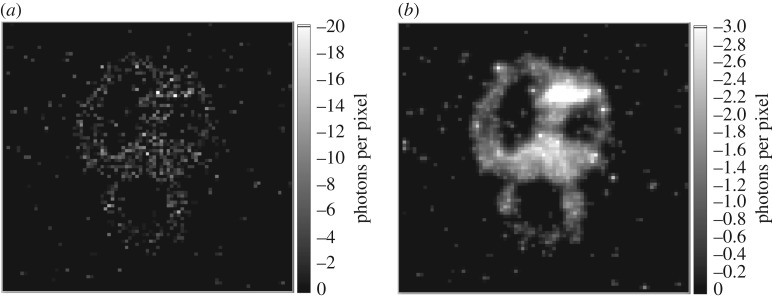
Photon-sparse images (*a*) are inherently noisy as a consequence of shot noise. Such images can be improved (*b*) by optimization under the assumption of pixel-to-pixel correlations, as described in the text.

There are many possible algorithms that can be applied for denoising images. Many of these algorithms assume Gaussian or arbitrary measurement noise models combined with desired image characteristics. For our typical images, the situation is somewhat different albeit more tightly defined. The measurements (i.e. number of photons) are inherently integer and subject to shot noise, whereas the underlying intensity is fractional. In the absence of any additional assumptions, then the reported image can only be that of the data itself. However, if one assumes a sparsity in the spatial frequency domain or similar smoothness constraint, then one can define a cost function from which an image can be reconstructed as a compromise between an image that satisfied the data (within statistical probabilities) and an image that satisfies the smoothness constraint [15,21] (figure 3).

## 7. Ghost imaging using classical correlations derived from a pseudo-thermal light source

The debate over whether ghost imaging based on photon pairs was uniquely quantum in nature inspired Gatti and co-workers [22] to both propose and demonstrate the use of pseudo-thermal light in place of the down-conversion source. Leaving aside the impact upon the quantum versus classical argument, their approach opened a new line of practical imaging techniques. The source of pseudo-thermal light is conveniently a laser passed through a rotating ground-glass screen and further spatially randomized by transmission through a turbid medium. The intensity cross-section of the resulting optical beam varies with time, and two near-identical copies of this beam are created using a beam splitter. Beyond the technical imperfection of the optics, the fidelity correlation between the two beams is limited only by their shot noise fluctuations; thus, for classical beams containing many photons, the strength of the correlation is high. In a similar manner to that of ghost imaging based on photon pairs, one of these classical beams is imaged onto the object and the other beam is imaged onto the detector. In the photon-pair system, the photon is measured to be at a specific position and the correlation between the two photons is zero or unity. In the many-photon classical system, the spatial measurement is a complicated pattern filling the field of view, and the strength of the correlation depends upon the degree of overlap between the pattern and the object, typically neither zero nor complete. These partial correlations mean that, when summed over all the random patterns, the resulting image contains the information of the object, yet this true image is superimposed upon a near-uniform, but non-zero background. Indeed, this presence of a non-zero background differentiates the many-photon ghost imaging using a thermal source from the background-free photon-pair ghost imaging using the parametric down-conversion source [10] discussed above.

Reconstructing the image from these measurements of partial correlations can be undertaken with various degrees of sophistication [23,24]. The simplest algorithm weights each of the known patterns by the magnitude of the signal recorded by the single-element detector and then sums these weighted patterns. The result approximates the image superimposed upon a near-uniform background. Improvements in signal-to-noise ratio can be achieved using various normalization techniques, and further improvements in signal-to-noise ratio and/or reduction in the number of required measurements can be achieved by applying the techniques of compressive sensing and inverse techniques.

## 8. Computational ghost imaging

This classical implementation of ghost imaging was taken one step further by Shapiro, who considered the case of using a spatial light modulator to create random intensity patterns with which to illuminate the object [25]. Because the optical patterns are derived from known programming of the spatial light modulator, the beam splitter and imaging detector can be eliminated from the apparatus. The spatial correlations are now not between two optical beams, but rather between one optical beam and a calculable pattern held in the computer memory. Hence this technique is known as computational ghost imaging. In this case, the only detector required is the single-element detector required to measure the intensity of the light that is transmitted/backscattered by the object. Again the image can be simply obtained by summing all of the patterns, each weighted by the signal from the detector.

This computational approach is extremely easy to implement since the spatial light modulator technology for shaping optical fields is quite advanced, largely as a result of display markets. The approach is particularly useful in spectral regions where the projection of a spatial pattern is technologically easier than imaging a scene. In this case, the only detector required is a single-element detector sensitive to the appropriate wavelength. Hence combining different detectors allows multi-modal imaging to give extended wavelength coverage and polarization sensitivity.

Another way of using multiple detectors is to position them so that they collect light backscattered in different directions. If the object itself is non-planar, then the magnitude of the backscattered light varies depending upon the orientation of the surface with respect to the detector positions. The result is that the reconstructed images appear as if they are illuminated with an off-axis light source, creating shadows and similar effects in the images [26]. Combining multiple images obtained with different detector positions and hence with different shadow details enables the three-dimensional form of the object to be reconstructed in a method referred to as photometric stereo.

## 9. Single-pixel camera

**Figure 4.**
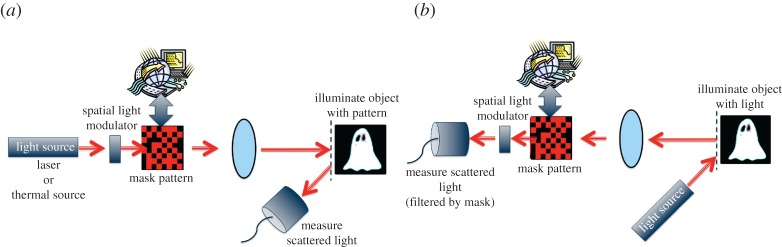
Computational ghost imaging (*a*) and single-pixel cameras (*b*) are similar in that they both reconstruct an image of the object from correlation measurements between the unknown object and the known masks.

The field of computational ghost imaging has a lot in common with classical computational imaging, in general, and with single-pixel cameras, in particular. Single-pixel cameras also use single-element detectors and spatial light modulators with which to produce an image. Whereas a computational ghost imaging system uses a spatial light modulator to spatially pattern the illumination light and a single-element detector to measure the light transmitted/backscattered from the object, a single-pixel camera uses unpatterned light to illuminate the object, a spatial light modulator to filter the projected image and a single-element detector to measure the correlation between the known pattern and the object [27] (figure 4).

Although the principles of computational ghost imaging/single-pixel camera can, in principle, be applied to complex fields, the availability of highly efficient and high-speed digital micro mirror (DMM) devices of the type found in nearly every presentation or digital projector makes working with intensity correlations alone very attractive. DMMs operate over broad spectral ranges and can display over 20 000 binary patterns every second. Selection of the optimum set of illumination patterns depends subtly on the characteristics of the objects being imaged. Ideally, the patterns are orthogonal to each other and share certain characteristics with those of the object. Naively, the number of patterns required is equal to the number of pixels in the image. However, although this estimate of the number of patterns required is correct for imaging objects that are themselves random, if the object is known to be sparse in a particular basis (e.g. spatial frequencies), then an excellent estimation of the image can be obtained by using far fewer patterns/measurements [28]. This form of compressed sensing is similar both in concept and in operation to the JPEG image compression algorithms central to picture and video transmission and storage.

**Figure 5.**
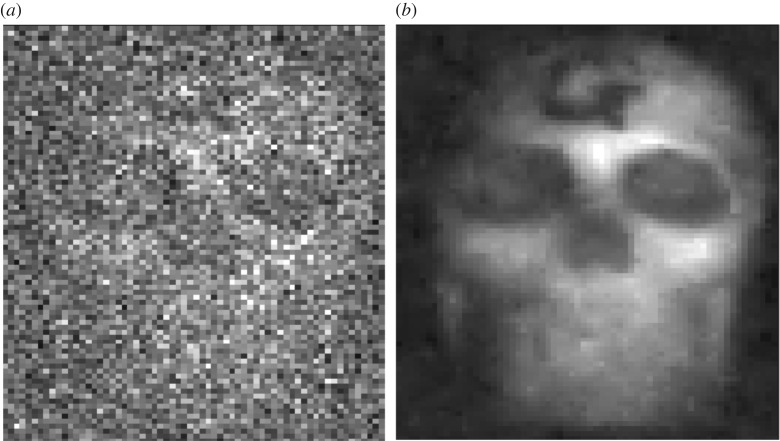
An image based on single-pixel camera data where the number of patterns measured is only 25% of the total pixel number (*a*) and an image derived from the same data but under the assumption of a minimum total second derivative of the pixel-to-pixel intensity (*b*).

The operation of these algorithms is possible through many different embodiments, but once again can be based upon the optimization of a cost function from which one obtains an image that is a compromise between the collected data (undetermined) and the assumption of spatial sparsity (figure 5) [29] and also, in the case of video, temporal sparsity.

The use of these single-element detectors to give image information gives certain opportunities not possible with low-cost focal-plane arrays. For example, the use of single-pixel cameras to create imaging systems in the short-wave infrared means that it is possible to image the attenuation of an appropriate laser diode, tuned to match the absorption of a target gas, such as methane. Beyond the coverage of hard-to-reach wavelengths [30,31], where detector arrays are either expensive or simply not available, single-element detectors have better temporal resolution, allowing depth ranging to be added to the imaging. Using pulsed illumination and time-resolved detection for each of the patterns gives not only *x*,*y* information associated with the image intensity but also *z* information for each image pixel [32,33].

## 10. Conclusion

Whether ghost imaging is a quantum effect, or merely a classical effect inspired by our quantum understanding, is an interesting debate, but perhaps more important is the question as to whether ghost imaging gives insights and techniques resulting in improved performance or even new modalities to imaging systems. Opportunities range from the options that quantum features bring to imaging at alternative and additional wavelengths to the ability to obtain images from the fewest number of photons limited only by orthogonality and sub-diffraction resolution. These options have significant impact on general laboratory instrumentation such as microscopy but also extend to commercial applications such as visualization of gas clouds and security considerations in covert imaging.

## Footnotes

1 Alternatively, the imaging can be set up such that the Fourier transform plane of the crystal is imaged both onto the object and onto the imaging detector (figure 2).

## Data accessibility

This article has no supporting data.
